# Indolent Abdominal Wall Round Cell Sarcoma With EWSR1-PATZ1 Fusion: A Case Report and Literature Review

**DOI:** 10.7759/cureus.89568

**Published:** 2025-08-07

**Authors:** Moreen Haddad, Victoria Xie, Justin Rivard, Miao Lu, Gregory J Garvin, Saman Kalikias, Yi Yan

**Affiliations:** 1 Department of Pathology, Max Rady College of Medicine, University of Manitoba, Winnipeg, CAN; 2 Interdisciplinary Health Program, Rady Faculty of Health Sciences, University of Manitoba, Winnipeg, CAN; 3 Department of Surgery, Max Rady College of Medicine, University of Manitoba, Winnipeg, CAN; 4 Department of Medical Imaging, St. Joseph’s Health Care London, London, CAN; 5 Department of Radiology, Max Rady College of Medicine, University of Manitoba, Winnipeg, CAN

**Keywords:** abdominal wall, ewsr1-non-ets fusions, ewsr1-patz1, prognosis, round cell sarcoma

## Abstract

Soft tissue sarcomas with Ewing Sarcoma Breakpoint Region 1-POZ/BTB and AT Hook Containing Zinc Finger 1 (EWSR1-PATZ1) gene fusion represent a recently recognized subgroup of "round cell sarcomas with EWSR1-non-ETS fusions." These tumors exhibit diverse morphologic features and a polyphenotypic immunoprofile, often co-expressing neural and skeletal muscle markers. Their clinical behavior ranges widely, from indolent to highly aggressive. We report the case of a 57-year-old man presenting with a 6.5 cm right abdominal wall mass incidentally discovered on CT imaging performed for choledocholithiasis. Ultrasound-guided biopsy revealed a low-grade spindle cell neoplasm, and the patient subsequently underwent marginal excision. Microscopic examination showed solid and pseudocystic architecture with thick fibrous septa, composed of round and spindle cells with eosinophilic or clear vacuolated cytoplasm and vesicular chromatin. Immunohistochemistry displayed positivity for CD99, BCL-2, and patchy desmin positivity. Targeted gene fusion analysis confirmed EWSR1-PATZ1 fusion. Given the long-standing, asymptomatic nature of the mass, the patient was managed with clinical surveillance and remains disease-free for three years post-excision. Due to the rarity of these tumors, their biologic behavior and optimal management remain uncertain, highlighting the need for individualized treatment strategies and long-term follow-up.

## Introduction

Soft tissue sarcoma with Ewing Sarcoma Breakpoint Region 1-POZ/BTB and AT Hook Containing Zinc Finger 1 (EWSR1-PATZ1) gene fusion is an emerging entity classified in the 5th edition of the WHO classification of soft tissue and bone tumors under “round cell sarcoma with EWSR1-non-ETS fusions” [1,2]. The EWSR1 gene is highly versatile, participating in fusion events with numerous partner genes across a range of mesenchymal and non-mesenchymal tumors. *PATZ1*, a member of the broad-complex, tramtrack and bric-à-brac-zinc finger (BTB-ZF) gene family, is involved in chromatin remodeling and transcriptional regulation. *EWSR1 *and *PATZ1 *are closely positioned on chromosome 22, approximately 2 Mb apart, and typically transcribed in opposite directions [2-5].

The clinical presentation of these uncommon tumors varies according to the available literature [6]. Histologically, these tumors typically consist of relatively uniform, cytologically bland round to spindled cells embedded in a fibromyxoid stroma, which often contains prominent fibrous bands, hyalinized blood vessels, and pseudoalveolar or microcystic spaces. Immunohistochemically, they usually exhibit a "polyphenotypic" profile, with co-expression of markers associated with skeletal muscle (e.g., desmin), epithelial cells (e.g., cytokeratin), Schwann cells (e.g., S100 protein), and glial cells (e.g., GFAP) [3,4,6-8].

The clinical behavior and prognosis of these tumors remain uncertain due to the limited number of reported cases [9]. In this case report, we present an indolent soft tissue EWSR1-PATZ1 round cell sarcoma arising in the abdominal wall. This contributes to expanding the histopathologic spectrum of this rare entity. Recognizing the variability within *EWSR1-PATZ1*-rearranged sarcomas is crucial to avoid misdiagnosis with other soft tissue tumors.

## Case presentation

**Figure 1:**
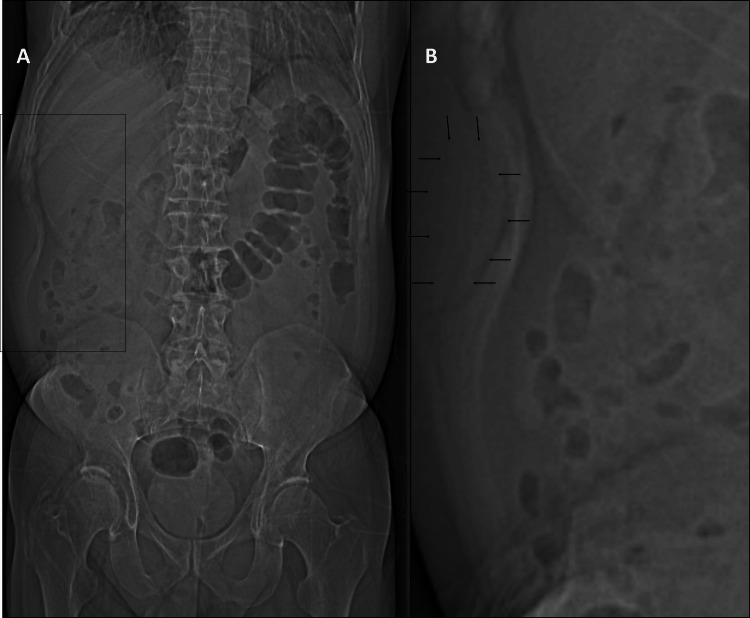
Scout image (abdominal radiograph from CT) of the abdominal wall mass. (A) The scout image demonstrated subtle soft tissue prominence along the right thoracoabdominal wall at the level of the right 12th rib, without evidence of underlying rib erosion or soft tissue calcification. (B) Magnified image highlighting the area of soft tissue prominence, suggestive of an underlying lesion (black arrows).

A 57-year-old gentleman presented with a right abdominal wall mass identified on CT imaging performed for jaundice and choledocholithiasis. Retrospectively, he had been aware of the mass for at least three years, with no significant change in size. He was otherwise asymptomatic. An abdominal CT scan revealed a 6.5 × 6.3 × 4.7 cm right abdominal wall mass. The scout image revealed a faint soft tissue prominence along the right thoracoabdominal wall, without evidence of rib erosion or soft tissue calcification (Figure 1).

**Figure 2:**
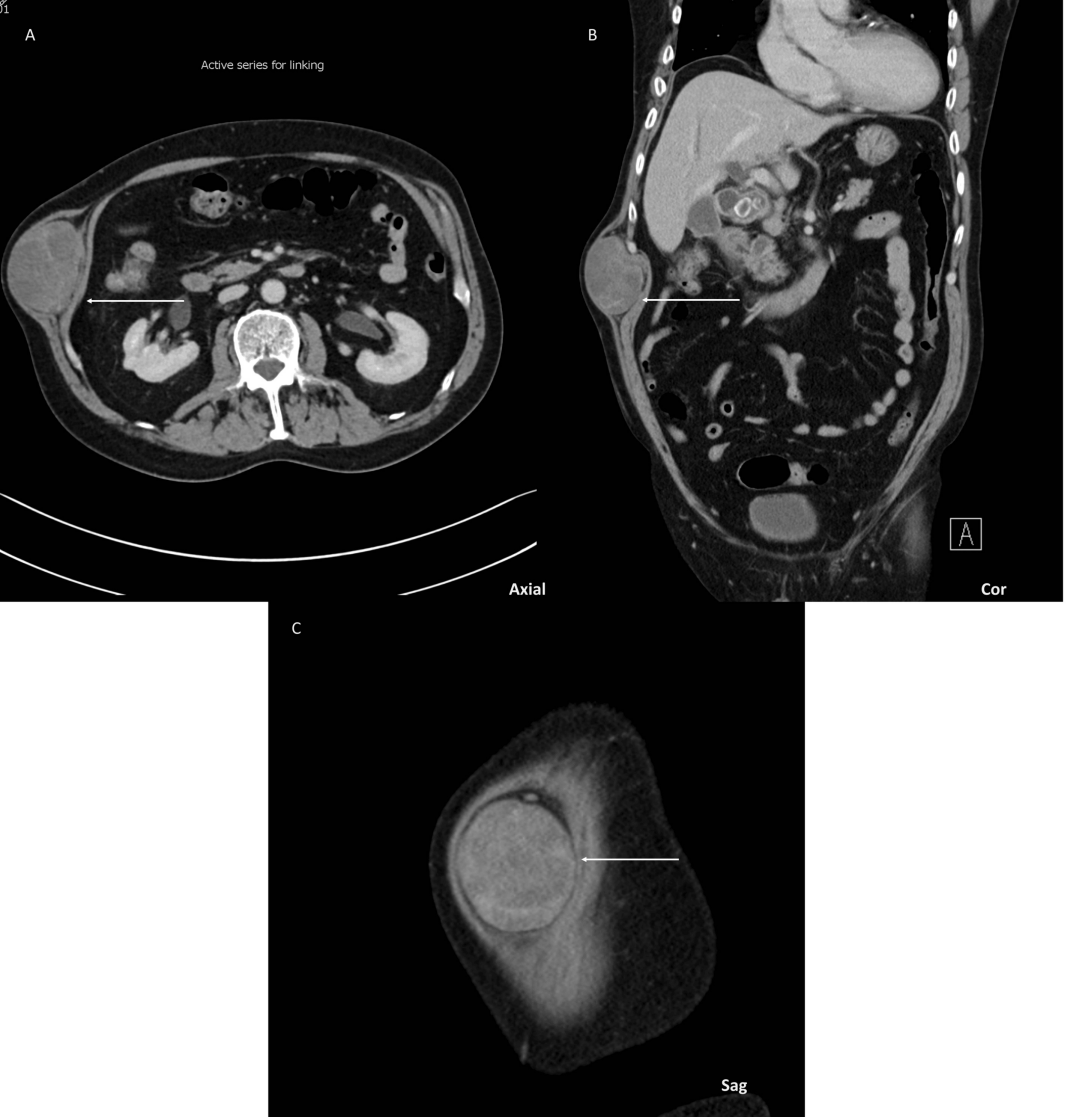
Abdominal/pelvic CT imaging of the abdominal wall mass. (A-C) Axial, coronal, and sagittal images of subsequent CT with soft tissue window showing a subcutaneous soft tissue mass along the right thoracoabdominal wall below the level of the right 12th rib. The mass demonstrated mixed density (arrows).

The mass was intramuscular, splaying the internal and external oblique abdominal wall muscles below the level of the 12th rib, best appreciated on axial and coronal images (Figure 2).

A mild associated mass effect was noted. Based on radiologic findings, the differential diagnosis included sarcoma, desmoid tumor, hematoma, or metastasis from an unknown primary.

**Figure 3:**
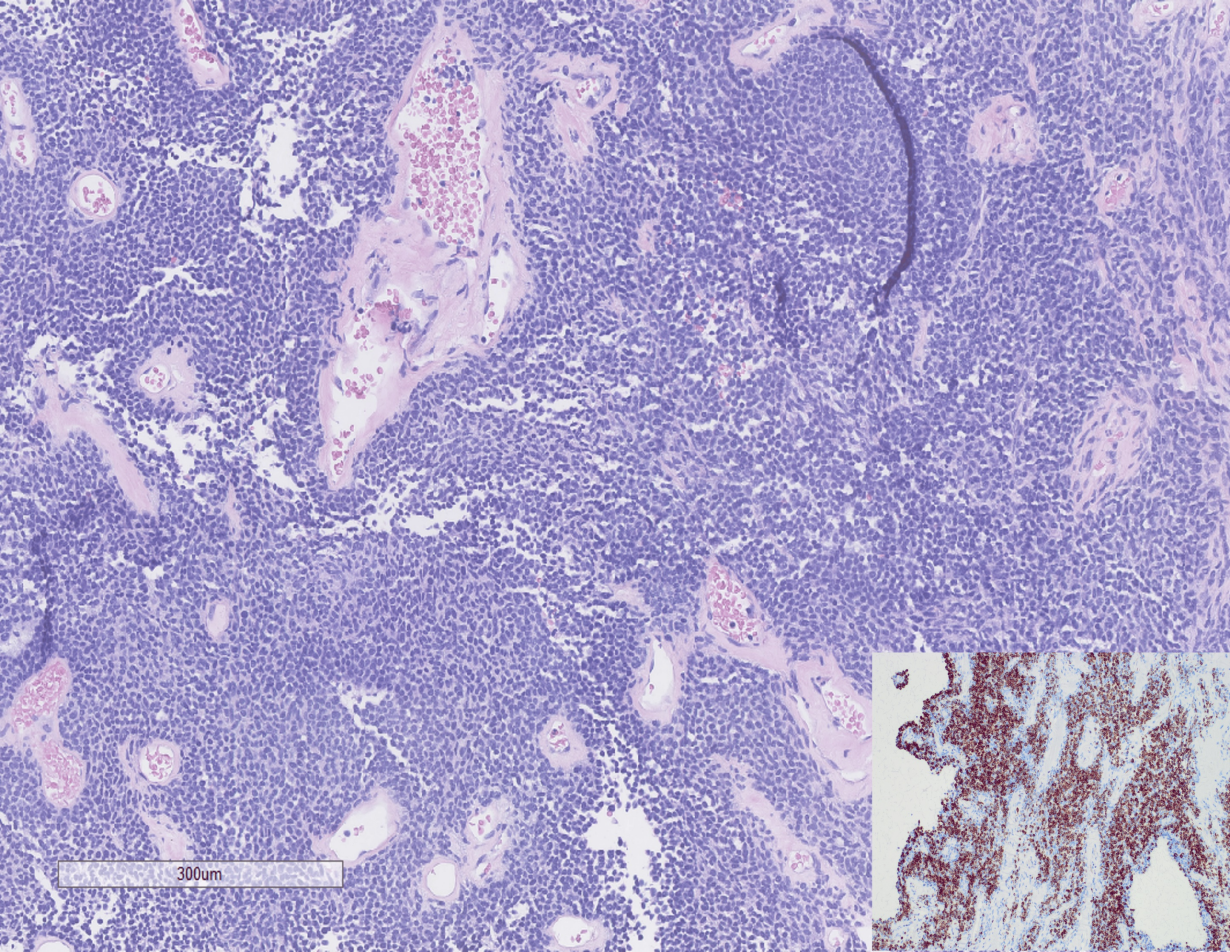
Histologic and immunohistochemical features of the biopsy specimen. The biopsy specimen revealed a hypercellular neoplasm composed of monotonous spindle cells with hyalinized blood vessels (10x). Inset: tumor cells showing patchy positivity for desmin.

Ultrasound-guided biopsy revealed a hypercellular neoplasm composed of monotonous spindle cells with elongated and oval nuclei and dense chromatin. Hemangiopericytoma-like vasculature with hyalinized vessel walls was present (Figure 3).

No necrosis or mitotic activity was identified. The initial differential diagnosis included solitary fibrous tumor, synovial sarcoma, and malignant peripheral nerve sheath tumor, among others. Immunohistochemistry showed positivity for CD99, BCL-2, and patchy desmin (Figure 3 inset). Rare cells showed weak positivity for CKAE1/AE3. The tumor was negative for STAT6, CD34, S100, SOX10, smooth muscle actin, EMA, myogenin, MyoD1, and WT1. The Ki-67 proliferation index was approximately 5%. Fluorescence in situ hybridization (FISH) was negative for rearrangement of the SS18 (18q11.2) locus. The biopsy was signed out as a low-grade spindle cell neoplasm, with a recommendation for complete excision.

The case was referred to a radiation oncologist to evaluate the potential benefit of neoadjuvant radiotherapy. An initial three-week course of radiation was considered. However, the surgeon presented the case at the Multidisciplinary Sarcoma Tumor Board. After expert review, the group determined that, in the absence of a definitive diagnosis of malignancy, preoperative radiation was not indicated. The consensus was to proceed with marginal excision to allow for definitive histologic evaluation. This approach aimed to avoid the morbidity of a full-thickness abdominal wall resection, reconstruction, and potential rib resection. If malignancy was confirmed on final pathology, a full-thickness thoracoabdominal wall resection could be considered.

**Figure 4:**
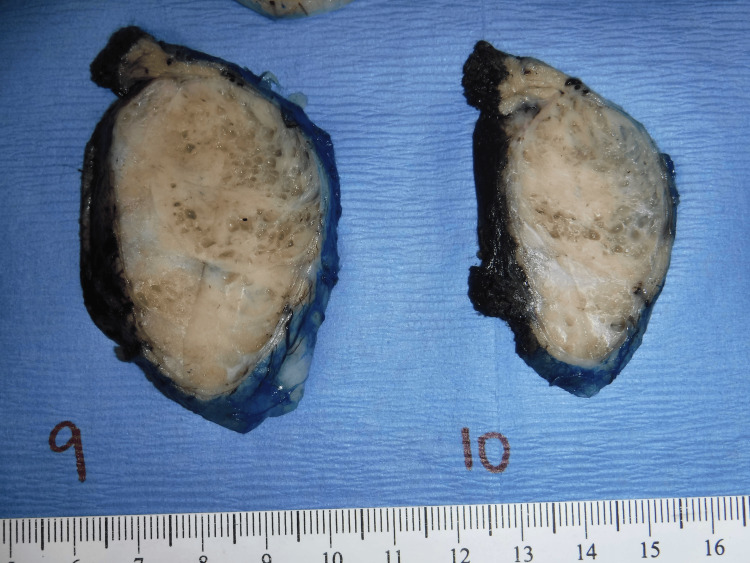
Gross image of the abdominal wall mass. The mass was well-circumscribed, gray-tan, and rubbery. The cut surface was solid with some cystic areas and fibrous septa.

Marginal excision of the mass was performed via circumferential dissection. The tumor separated easily from surrounding musculature, except on the deep side, where it appeared partially fixed to the underlying abdominal wall. Therefore, muscle fibers were included in the specimen at the deep margin. Grossly, the mass was well-circumscribed, gray-tan, and rubbery, measuring 6.7 × 6.0 × 4.8 cm and weighing 103 g. It was submitted with the attached portion of the abdominal wall. The cut surface was predominantly solid, with areas of cystic change and fibrous septation (Figure 4).

**Figure 5:**
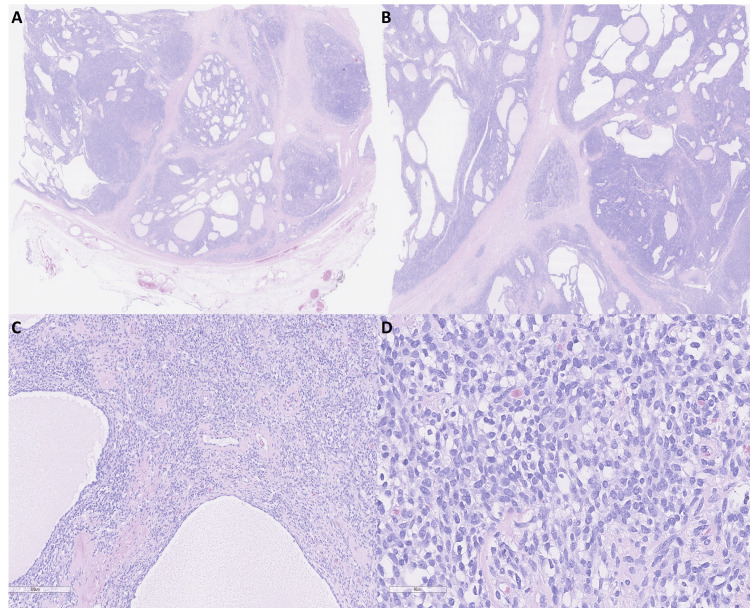
Histopathologic features of the excised mass. (A, B) Low-power views showing a thick fibrous capsule extending inward and dividing the tumor into nests and nodules (0.4x). (C) Numerous hyalinized vessels and pseudoalveolar spaces containing proteinaceous material (10x). (D) High-power view showing sheets of round to spindled cells with indistinct cell borders, eosinophilic to clear cytoplasm, vesicular chromatin, and occasional small nucleoli (40x).

Microscopically, the tumor was circumscribed and surrounded by a thick fibrous capsule that extended inward to divide the tumor into nests and nodules (Figures 5A, 5B). Pseudoalveolar spaces containing proteinaceous material and numerous hyalinized vessels were observed (Figure 5C). The solid areas were composed of sheets of round and spindle cells with indistinct borders, eosinophilic to clear vacuolated cytoplasm, vesicular chromatin, and occasional small nucleoli (Figure 5D).

Tumor necrosis and mitoses were absent. The resection specimen demonstrated identical immunohistochemical features to the biopsy specimen. Next-generation sequencing (performed at Mayo Clinic, Rochester, MN) targeting 138 genes, including those relevant to the major differential diagnoses such as BCOR, CIC, and SS18, identified an *EWSR1-PATZ1* gene fusion (data not shown).

Given the long-standing nature of the mass and its indolent features, it likely represents a tumor with nonaggressive behavior. Although re-resection could be considered due to the close margins, it would require a radical thoracoabdominal wall resection including resection of adjacent ribs. After the discussion, the sarcoma team recommended continued close surveillance. Plans were made for follow-up imaging, including MRI and CT of the chest, abdomen, and pelvis in six months, followed by a comprehensive clinical review with the patient. The patient has remained free of disease for three years following excision.

## Discussion

Round cell sarcomas featuring EWSR1-non-ETS fusions represent a rare subset of soft tissue tumors. These sarcomas are defined by genetic rearrangements involving EWSR1 or FUS fused to non-ETS partners, in contrast to classic EWSR1-ETS fusions typically associated with Ewing sarcoma [6,10]. The PATZ1 gene encodes a zinc finger protein with tumor-suppressor functions and is located in close proximity to EWSR1 on chromosome 22 [9]. When EWSR1 and PATZ1 fuse in-frame, the resulting fusion protein lacks the transcriptional repressor domain of PATZ1, converting it into a transcriptional activator [9].

EWSR1-PATZ1 sarcomas can arise in the deep soft tissues of the chest or abdominal wall and have been reported across a wide age range [3,4,11]. A few cases have also been documented in the head and neck region as well as the central nervous system [12-14].

While approximately seven cases of EWSR1-PATZ1 sarcomas in the abdominal wall have been reported (Table 1), their imaging characteristics remain poorly defined. The CT findings in our case, similar to those in a previous case report [4], showed a well-circumscribed, heterogeneous intramuscular soft tissue mass. However, the features are nonspecific and can mimic other abdominal wall lesions, such as desmoid tumors, hematoma, or metastasis.

**Table 1:** Clinical features of abdominal wall EWSR1-PATZ1 sarcomas. M: male, F: female, N/A: not available, Sx: surgery, RT: radiation therapy, ANED: alive without evidence of disease, AWD: alive with disease, mets: metastasis.

Case	Age	Sex	Size (cm)	Imaging	Treatment	Follow-up	References
1	50	M	6.7	N/A	None	ANED (2.5 m)	Dehner 2024 [11]
2	40	M	6.2	Circumscribed, isointense to the adjacent muscle (CT scan)	None	ANED (77 m)	Warmke and Meis [4]; Dehner et al. [11]
3	44	M	4	N/A	Sx	ANED (19 m)	Michal et al. [3]
4	36	M	7	N/A	Sx	N/A	Michal et al. [3]
5	49	M	5.8	N/A	Sx	N/A	Michal et al. [3]
6	46	F	2.5	N/A	Sx + RT	Multiple lung mets (36 and 48 m), AWD (60 m)	Michal et al. [3]
7	66	M	2.5	N/A	N/A	Lung mets at presentation	Michal et al. [3]

These tumors manifest as solid-cystic masses and exhibit a range of histological features and immunophenotypic profiles (Table 2).

**Table 2:** Pathologic features of abdominal wall EWSR1-PATZ1 sarcomas Myogenic markers (desmin, myogenin, myoD1, SMA, caldesmon) and neurogenic markers (e.g., S100, SOX10, GFAP) are in bold.

Case	Macro	Micro	Immunohistochemistry	Fusion	References
1	N/A	N/A	*Positive*: S100, desmin, myoD1, myogenin, keratins, CD99. *Negative*: CD34	EWSR1 exon 8–PATZ1 exon 1	Dehner et al. [11]
2	Circumscribed, no hemorrhage or necrosis	Low-grade spindle/round cells, rhabdomyoblasts	*Positive*: S100, SOX10, desmin, myoD1, myogenin, GFAP. *Negative*: keratins	EWSR1 exon 8–PATZ1 exon 1	Warmke and Meis [4]; Dehner et al. [11]
3	N/A	Low-grade spindle/round cells	*Positive*: S100, GFAP, myoD1, PAX7, WT1 (cytoplasmic), MDM2, AE1/3. *Negative*: desmin, myogenin, SOX10, CD99, synaptophysin, CD34, etc.	EWSR1 exon 8–PATZ1 exon 1	Michal et al. [3]
4	N/A	Low-grade spindle/round cells	*Positive*: S100, SOX10, GFAP, desmin, myoD1, PAX7, WT1 (cytoplasmic), AE1/3, CD34 (weak). *Negative*: myogenin, CD99, synaptophysin, SMA, STAT6, etc.	EWSR1 exon 8–PATZ1 exon 1	Michal et al. [3]
5	N/A	Low-grade spindle/round cells	*Positive*: S100, GFAP, desmin, MyoD1, myogenin, SMA, WT1 (cytoplasmic), CD34. *Negative*: AE1/3, other keratin	EWSR1 exon 8–PATZ1 exon 1	Michal et al. [3]
6	N/A	Round cell	*Positive*: GFAP, desmin, myoD1, caldesmon, CD99. *Negative*: S100, myogenin, AE1/3, SOX10, WT1, CD34, etc.	EWSR1 exon 8–PATZ1 exon 1	Michal et al. [3]
7	N/A	High-grade spindle/round cell	*Positive*: desmin, myoD1, myogenin, AE1/3, CD99. *Negative*: S100, GFAP, PAX7	EWSR1 exon 8–PATZ1 exon 1	Michal et al. [3]

Tumor cells typically appear as small, round, and/or spindled cells, often surrounded by fibrous stroma. The presence of necrosis and mitotic figures can vary [11]. Immunophenotypically, they display a polyphenotypic profile, with inconsistent co-expression of myogenic markers (e.g., desmin, myogenin, myoD1) and neurogenic markers (e.g., S100, SOX10, GFAP) [8,9]. In our case, patchy desmin staining was present, but there was no expression of neurogenic or other myogenic markers, highlighting the tumor’s polyphenotypic nature [8].

The differential diagnosis may include a wide range of tumors with overlapping features, such as BCOR- or CIC-rearranged sarcomas, solitary fibrous tumor, desmoplastic small round cell tumor, Ewing sarcoma, myoepithelioma, rhabdomyosarcoma, and malignant peripheral nerve sheath tumor [3].

Similar to our case, a prolonged history prior to clinical presentation has been reported in several instances, reflecting the indolent behavior of some EWSR1-PATZ1 sarcomas [7,9]. However, other cases have shown aggressive clinical courses, including locoregional or distant metastases and death within 5-32 months of diagnosis [9,13]. Non-infiltrative growth, low-grade spindle/round cell morphology, and a low mitotic count seem to be associated with a better prognosis [3]. These features are all present in our case, consistent with the favorable follow-up. Some studies have identified secondary driver mutations, particularly involving CDKN2A, in a subset of these tumors, correlating with a poorer prognosis [3,9]. While we did not assess CDKN2A status in our case, the tumor’s prolonged indolent course suggests a low likelihood of such a mutation.

The optimal treatment approach for EWSR1-PATZ1 sarcomas remains undefined. Reported cases have demonstrated resistance to systemic chemotherapy. Given their malignant potential, at least complete surgical excision is recommended, followed by ongoing clinical surveillance [3,8,9,11].

## Conclusions

In summary, round cell sarcomas with EWSR1-PATZ1 fusions represent a rare and heterogeneous group of tumors with diverse histological and immunophenotypic features across various anatomical sites. In the absence of molecular confirmation, these tumors can be particularly difficult to diagnose due to their overlap with other small round cell neoplasms. Their clinical behavior remains incompletely understood, and longitudinal data collection is needed to better stratify patients by risk. While surgical resection with long-term follow-up remains the cornerstone of management, further studies are essential to clarify their biology and guide the development of more effective diagnostic and therapeutic strategies.
